# Bacterial lipopeptides from *Bacillus*: natural biostructuring agents for improving texture and stability in tomato-based functional foods

**DOI:** 10.3389/fnut.2026.1830310

**Published:** 2026-06-08

**Authors:** Houda Gharsallah, Rose Daphnee Tchonkouang, Aymen Ben Mabrouk, Najeh Jaoued, Ozan Tas, Sami Boufi, Maria Margarida Cortez Vieira, Mecit Halil Oztop, Zied Zarai

**Affiliations:** 1 Departement of Food Technology, High Institute of Biotechnology of Sfax, University of Sfax, Sfax, Tunisia; 2 MED – Mediterranean Institute for Agriculture, Environment and Development and CHANGE – Global Change and Sustainability Institute, Universidade do Algarve, Campus de Gambelas, Faro, Portugal; 3 LMSE, Faculty of Science, University of Sfax, Sfax, Tunisia; 4 National Institute of Research and Physicochemical Analysis (INRAP), Biotechnopole of Sidi Thabet, Ariana, Tunisia; 5 Faculty of Engineering, Food Engineering, Middle East Technical University, Ankara, Türkiye; 6 Department of Food Engineering, Higher Institute of Engineering, Campus da Penha, Universidade do Algarve, Faro, Portugal; 7 Laboratory of Biochemistry and Enzymatic Engineering of Lipases, ENIS, University of Sfax, Sfax, Tunisia

**Keywords:** *Bacillus*, bacterial lipopeptides, functional foods, natural bio-structuring agents, rheology, tomato-based products

## Abstract

**Introduction:**

Recent advances in functional food formulation have highlighted bacterial lipopeptides as natural biomolecules capable of replacing synthetic additives while enhancing the physicochemical properties of food systems. Their amphiphilic structure makes them promising candidates for improving texture, stability, and emulsification in complex matrices such as tomato-based products.

**Methods:**

In this study, lipopeptides produced by Bacillus strains were structurally characterized using ^1^H-NMR and two-dimensional ^1^H–^1^H COSY spectroscopy to confirm their amphiphilic nature. Tomato-based formulations (juice and sauce) were enriched with lipopeptides at concentrations of 0.5, 1.0, and 2.0 g/L. Rheological behavior was evaluated under steady-shear conditions to assess flow properties and time-dependent structural changes.

**Results:**

Spectroscopic analysis confirmed the presence of characteristic aliphatic fatty acid chains and peptide residues, validating the lipopeptidic structure. All formulations exhibited shear-thinning behavior; however, the addition of lipopeptides significantly influenced time-dependent rheological properties. Improved texture, viscoelasticity, and stability were observed, with optimal performance achieved at low-to-moderate concentrations (H6: 0.5–1 g/kg; S15: up to 2 g/kg). Higher concentrations led to partial network disruption and reduced system stability.

**Discussion:**

These findings demonstrate that bacterial lipopeptides act as effective natural bio-structuring agents in tomato-based products. Their ability to enhance microstructural integrity and emulsification without compromising stability at optimal concentrations highlights their potential as sustainable alternatives to synthetic additives in functional food development.

## 1 Introduction

The development of new food formulations remains a central focus in modern food processing. The success and acceptance of such innovations largely depend on their composition, which is determined by the judicious selection of ingredients. The food industry constantly strives to develop, improve, or modify products while minimizing production costs and processing time (1).

Tomato is among the most widely cultivated vegetables worldwide and a key raw material for industrial products such as juice, purée, paste, ketchup, and sauces, used either directly or in further formulations (2, 3).

These products are complex dispersions of pulp particles in an aqueous phase, and their rheological behavior is strongly influenced by composition and processing conditions. Rheological characterization is therefore essential for process design, optimization, and quality control. Tomato-based systems commonly exhibit non-Newtonian behavior, including shear-thinning and viscoelastic properties, requiring advanced rheological approaches (4).

Non-Newtonian fluids can be classified as time-independent, time-dependent, or viscoelastic, and are described using parameters such as viscosity, shear stress, shear rate, and elasticity. Oscillatory rheology captures both viscous and elastic responses, whereas steady shear mainly reflects viscosity. These properties are closely linked to microstructure and molecular organization.

Viscosity and color are major determinants of consumer acceptance, with thick texture and intense red color being preferred (5). To achieve these attributes, functional ingredients are used to improve texture, stability, and color retention. Acid and salt reduce enzymatic browning, while hydrocolloids such as gelatin and flour enhance viscosity and consistency (6, 7), also affecting water mobility and product stability.

Rheology provides key insights into structure–function relationships in tomato products (8). Consistency in semi-solid systems such as sauces and pastes depends mainly on particle concentration and soluble polysaccharides, including pectin and hemicelluloses, which contribute to viscosity and structural stability (9, 10). However, increasing demand for clean-label products has driven interest in replacing conventional additives with natural alternatives.

Among emerging bio-based ingredients, bacterial lipopeptides and other biosurfactants have attracted considerable attention due to their biodegradability, low toxicity, and multifunctional properties, positioning them as promising alternatives to synthetic food additives (11–13). Predominantly produced by *Bacillus* species, these amphiphilic molecules exhibit strong surface activity that enables efficient reduction of interfacial tension, stabilization of oil-in-water emulsions, and improvement of dispersion in complex food matrices, even at low concentrations (12). Beyond their emulsifying function, they play a key structural role in food systems by interacting with proteins, polysaccharides, and lipids, thereby promoting interfacial film formation, enhanced network organization, and improved viscoelastic properties (14). At the microstructural level, they contribute to reduced droplet size distribution and reinforcement of the continuous phase, which collectively improves rheological behavior and long-term physical stability of emulsified systems (11). Among them, surfactin produced by *Bacillus subtilis* has been extensively studied and shown to exhibit emulsifying performance comparable to conventional synthetic surfactants while simultaneously enhancing structural integrity of formulated products (15). In addition, biosurfactants have been associated with antimicrobial and antioxidant activities, which further contribute to food preservation and shelf-life extension (16). Their compatibility with clean-label requirements, combined with their multifunctional technological and bioactive properties, highlights their strong potential for the design of next-generation functional food systems (13).

Among these, biosurfactants such as lipopeptides have attracted attention due to their amphiphilic nature, which allows them to alter the rheological and interfacial characteristics of food matrices. *Bacillus* species produce lipopeptides, which are capable of improving food texture, stability, and shelf life due to their surface active, emulsification, and bioactive properties (17–19).

This study investigates the impact of lipopeptide supplementation on the rheological and physicochemical properties of tomato juice and sauce. Lipopeptides are incorporated at increased concentrations to assess their influence on viscosity, shear-thinning behavior, time-dependent flow, color, water status indicators, and proton molecular mobility. The findings aim to support the development of innovative tomato-based formulations with enhanced texture, stability, and sensory attributes by utilizing lipopeptide biosurfactants as natural and multifunctional ingredients.

## 2 Materials and methods

### 2.1 Functional tomato juice and sauce preparation

Fully ripe high-lycopene “H1657” tomatoes from Ribatejo, Portugal, were used as raw material. The preparation of ingredients and formulation of functional tomato products were carried out following the protocols described by Tchonkouang et al. (20). Tomato fruits were peeled and processed into pulp using a Thermomix TM6-1 (Vorwerk, Elektrowerke GmbH & Co. KG, Wuppertal, Germany). Tomato peel powder (TPP) was produced by drying peels at 50 °C for 24 h in a U50 oven (Memmert GmbH + Co. KG, Büchenbach, Germany) and milled using a centrifugal mill ZM1 (Retsch GmbH, Haan, Germany). Olive powder (OP) was prepared by blending pitted olives with water, followed by high-pressure homogenization at 1,000 bars using a Panda Plus homogenizer (GEA Niro Soavi, Parma, Italy). The slurry was freeze-dried (Liyolife, FD5CT, İstanbul, Türkiye) and milled with the same centrifugal mill ZM1 (Retsch GmbH, Haan, Germany). Commercially sourced 100% vegan pea protein powder (PP) was obtained from Vegrano (Türkiye). The pulp destined to produce the sauce was subjected to a hot break pretreatment that involved heating the tomato pulp at 85 °C in a water bath and immediately transferring it to an ice water bath to halt the hot break process. The pulp used to prepare the juice was treated using a cold break process at 65 °C. In addition, a portion of the cold-break pulp was centrifuged at 4,500 RPM for 5 min, and the supernatant (liquid extract of the pulp) was reserved. Two functional tomato-based products were formulated: a juice and a sauce. The juice was prepared using 26% tomato pulp and 70% liquid extract of the pulp, with the addition of 1% tomato peel powder, 1% freeze-dried olive powder, 1% pea protein, and 1% salt. In contrast, the sauce formulation consisted mainly of tomato pulp (93.06%) and included 3.47% tomato peel powder, 1.66% freeze-dried olive powder, and 1.82% pea protein. Neither salt nor liquid extract was incorporated into the sauce. The mixtures were homogenized using a hand blender and transferred to the HPH (Panda Plus, GEA Niro Soavi, Parma, Italy), for further homogenization at pressures of 50–100 bars (juice) and 200–500 bars (sauce).

### 2.2 Incorporation of lipopeptides in formulated products

The production and extraction of *Bacillus velezensis* (H6) (access number: PQ539041) and *Bacillus subtilis* (S15) (access number: PQ541200) derived lipopeptides were performed following the optimized methodology of Gharsallah et al. (21), aimed at maximizing yield and maintaining bioactivity for food applications. Liquid chromatography–tandem mass spectrometry (LC–MS/MS) revealed diverse lipopeptide families, including surfactins, iturins, and fengycins, with strain H6 producing the highest concentrations (21). The biochemical characterization demonstrated that the biosurfactant extracts exhibited exceptional emulsifying and foaming properties, with remarkable stability, achieving up to 90% emulsification and foaming activity (21).

Lipopeptides (H6 and S15) were incorporated into the formulations at concentrations of 0.5, 1, and 2 g/L. The mixtures were briefly homogenized using a hand blender to ensure uniform dispersion of lipopeptides. Control samples without lipopeptides were also prepared for comparison. All formulations were stored at 4 °C for 24 h before further analysis to allow stabilization of the bioactive compounds. Rheological measurements and physicochemical characterization were conducted immediately after storage.

### 2.3 Nuclear magnetic resonance spectroscopy analysis

^1^H NMR spectra of the lipopeptides were recorded at 500.13 MHz using a Bruker Avance III 500 UltraShield Plus spectrometer operating at 298 K, equipped with a 5 mm BBFO probe. Samples were dissolved in 100% Chloform (CDCl_3,_ Sigma Aldrich) at a concentration of 30 mg/mL. Each spectrum was acquired using 256 scans, a spectral width of 8,012 Hz (16 ppm), an acquisition time of 4.0 s, a recycle delay of 2.0 s, and a 90° pulse angle, yielding a total duration of approximately 13 min per sample, in accordance with standard high-resolution NMR acquisition protocols (22).

Two-dimensional ^1^H–^1^H correlation spectroscopy (COSY) was carried out using the COSYPHPR pulse program from the Bruker library with the following parameters: a 90° pulse width of 9.8 μs, a spectral width of 4,800 Hz (yielding an acquisition time of 0.2 s), a mixing time of 800 ms, and a relaxation delay of 1 s, following established methodologies for 2D NMR experiments and structural elucidation of complex organic molecules (22, 23). These experimental conditions are consistent with previously reported NMR studies for the structural characterization of lipopeptide biosurfactants, including surfactin and related compounds (24, 25).

### 2.4 Effect of lipopeptides on the tomato juice microstructure

The impact of lipopeptide incorporation on the microstructure of tomato products was assessed using optical inversed microscopy. Microstructural analysis was performed on samples containing 0.5, 1, and 2 g/L of lipopeptides, as well as on control samples without lipopeptides.

The microstructure of the treated and control tomato products samples was examined using an inverted optical microscope (model, brand). Images were captured under brightfield conditions at various magnifications (10×, 20×, and 40×). Microstructural changes such as particle aggregation, network formation, and phase separation were visually assessed and documented.

### 2.5 Rheological measurements

Rheological measurements were conducted using a Kinexus Pro+ rheometer (Malvern Instruments, United Kingdom). For all samples, tests were performed in dynamic mode at a temperature of 25 °C using a plate-plate geometry (rough surface, diameter: 20 mm, gap thickness = 0.4 mm). The linear domain was initially determined by sweeping the storage modulus (G′) and loss modulus (G′′) versus strain, ranging from 0.01 to 100%. Subsequently, a frequency sweep was performed at a fixed strain within the elastic linear domain.

### 2.6 Viscosity measurements

Steady-State Measurement Flow test was conducted under steady state flow, using spindles 18 and 34 in a viscometer Brookfield Ametek DV2T Mil (Massachusetts, United States) in a shear rate range of 0.1–100 s^−1^, and with temperatures ranging from 25 to 40 °C.

The flow behavior of the sauces was modeled using the Bingham and Hershel–Bulkley model (Equation 1) where SS is the shear stress (Pa), *k_ref_* is the consistency index (Pa.s^n^), ˙SR is the shear rate (s^−1^), and *n* is the flow behavior index (dimensionless).

### 2.7 Mathematical modeling

According to their behavior, fluids are classified as “Newtonian or non-Newtonian.” A Newtonian fluid is independent of shear rate, always presenting the same viscosity at constant pressure and temperature, being explained by a single parameter rheological model (Equation 1), where 𝜏 denotes shear stress, *μ* refers to the Newtonian fluid’s viscosity, and 𝛾 denotes the shear rate. Using this correlation, a Newtonian material can be described by a straight line if the shear rate is plotted against shear stress.

SS=μSR (1)

When fluid is non-Newtonian, the Power law (or Ostwald-de Waele) fluid model (Equation 2) fits very well with the experimental data:

$$\mathrm{SS}=K{\left(\mathrm{SR}\right)}^{n}$$ (2)

Where K is the consistency index, and *n* may assume several values. If *n* < 1, the fluid is called pseudoplastic; when *n* = 1, the fluid shows Newtonian behavior; when *n* > 1, the fluid is called dilatant.

In this situation, the viscosity *μ* depends on the shear rate and therefore it is called apparent viscosity *μ_app_*, Equation (3)

$$\mu_{\mathrm{app}}=K\;SR^{n-1}$$ (3)

$$\mathrm{SS}=k\;\mathrm{SR}+SS_{0}$$ (4)

Some fluids behave like Newtonian fluids, but only when the material’s shear stress overcomes the apparent yield stress. Overall, such fluids can be described by the Bingham plastic model (Equation 4):

When these fluids are non-Newtonian, the Herschel-Bulkley fluid model (a viscoplastic model obtained by joining the Bingham plastic model and power Law) (Equation 5) usually fits well to the experimental data (26), where $SS_{o}$ is the yield stress, “ k ” “ n ” are constant and < 1.

$$\mathrm{SS}=k{\left(\mathrm{SR}\right)}^{n}+SS_{o}$$ (5)

The Bingham Plastic model describes *η*, the apparent viscosity of the fluid as Equation 6:

$$\mu_{\mathrm{app}}=\frac{SS_{0}}{\mathrm{SR}}+\mu_{\infty }$$ (6)

Where $\mu_{\infty }$ is the plastic viscosity or the coefficient of rigidity.

The Herschel-Bulkley model describes *μ*, the apparent viscosity of the fluid as Equation 7:

$$\mu_{\mathrm{app}}=\frac{SS_{0}}{\mathrm{SR}}+K{\left(\mathrm{SR}\right)}^{n-1}$$ (7)

When evaluating the dependence of SS on temperature, the Arrhenius equation often applies (Equation 8) where $\;K_{\mathrm{ref}}$ is the consistency index at the reference temperature T_ref_ (in Kelvin), K is the consistency index at temperature T, *Ea* is the activation energy, usually expressed in kJ or kcal per mole, and R is the Universal gas constant in corresponding units (4).

$$K=K_{\mathrm{ref}}\;\exp^{-\frac{E_{a}}{R}(\frac{1}{T}-\frac{1}{T_{\mathrm{ref}}})}$$ (8)

Therefore, both the Bingham and the Herschel-Bulkley fluid models can be written as (Equations 9and 10),

$$\mathrm{SS}=K_{\mathrm{ref}}\exp^{-\frac{E_{a}}{R}(\frac{1}{T}-\frac{1}{T_{\mathrm{ref}}})}\mathrm{SR}+SS_{o}$$ (9)

$$\mathrm{SS}={\left(K_{\mathrm{ref}}\exp^{-\frac{E_{a}}{R}(\frac{1}{T}-\frac{1}{T_{\mathrm{ref}}})}\right)}^{n}\mathrm{SR}+SS_{o}$$ (10)

and the effect of temperature on the apparent viscosity app can be predicted by Equations 11 or 12, depending on the model being Bingham or Herschel Buckley, respectively.

$$\mu_{\mathrm{app}}=\frac{SS_{0}}{\mathrm{SR}}+\mu_{\text{plastic}}$$ (11)

And

$$\mu_{\mathrm{app}}=\frac{SS_{0}}{\mathrm{SR}}+K_{\mathrm{ref}}\exp^{-\frac{E_{a}}{R}(\frac{1}{T}-\frac{1}{T_{\mathrm{ref}}})}{\left(\mathrm{SR}\right)}^{n-1}$$ (12)

### 2.8 Measurements of color

The CIELab parameters (L*, a*, b*) were directly read with a spectrophotocolorimeter MS/Y-2500 (Hunterlab, In., Reston, VA, United States), calibrated with a white tile. In this coordinate system, the L* value is a measure of lightness, ranging from 0 (black) to 100 (white); the a* value ranges from − 100 (green) to + 100 (red) and the b* value ranges from − 100 (blue) to + 100 (yellow).

### 2.9 Statistical analysis

All experiments were performed in triplicate (*n* = 3), and results are expressed as mean ± standard deviation. Statistical analysis was carried out using IBM SPSS Statistics software (IBM Corp., Armonk, NY, USA). Prior to analysis, data normality was verified. One-way analysis of variance (ANOVA) was applied to evaluate the effect of lipopeptide type (H6 and S15) and concentration (0.5, 1, and 2 g/L) on the physicochemical, rheological, and microstructural properties of tomato juice and sauce. When significant differences were detected, means were compared using Tukey’s *post-hoc* test. Differences were considered statistically significant at *p* < 0.05.

## 3 Results and discussion

### 3.1 NMR spectroscopy

Nuclear Magnetic Resonance (NMR) spectroscopy, particularly one-dimensional proton (^1^H NMR) recorded in deuterated chloroform (CDCl₃), was employed as a key analytical tool to elucidate the molecular structure of lipopeptides extracted from *Bacillus velezensis* (H6) and *Bacillus subtilis* (S15).

The chemical structure of the lipopeptides present in the biosurfactant mixture was confirmed using 1H NMR spectroscopy. The chemical shift observed at 0.86 ppm indicates the presence of terminal methyl groups (–CH₃) in fatty acid acyl chains, as well as methyl groups attached to amino acids, confirming the amphiphilic nature of the molecules (27–30).

A signal at 1.26 ppm corresponds to methylene groups in the aliphatic chain –(CH₂)ₙ–, while the presence of a Me_2_CH– group is evidenced by a signal at 1.6 ppm. Chemical shifts between 2.0 and 2.5 ppm are assigned to allylic –CH_2_ and *α*-CH_2_ protons.

The spectral region between 3.2 and 4.3 ppm was attributed to glucose ring protons, methoxy groups (–OCH₃), and backbone peptide protons, including H*α* and H*β* in moieties such as –CH₂COO^−^, –OCH–, and –COOCH–. Notably, signals at 4.9 ppm and 5.2 ppm correspond to protons within esterified and peptide environments.

Furthermore, the spectrum confirmed the presence of amide protons (NH) and olefinic protons associated with unsaturated fatty acid chains (see Figure 2), supporting the structural features of the biosurfactant.

**Figure 1:**
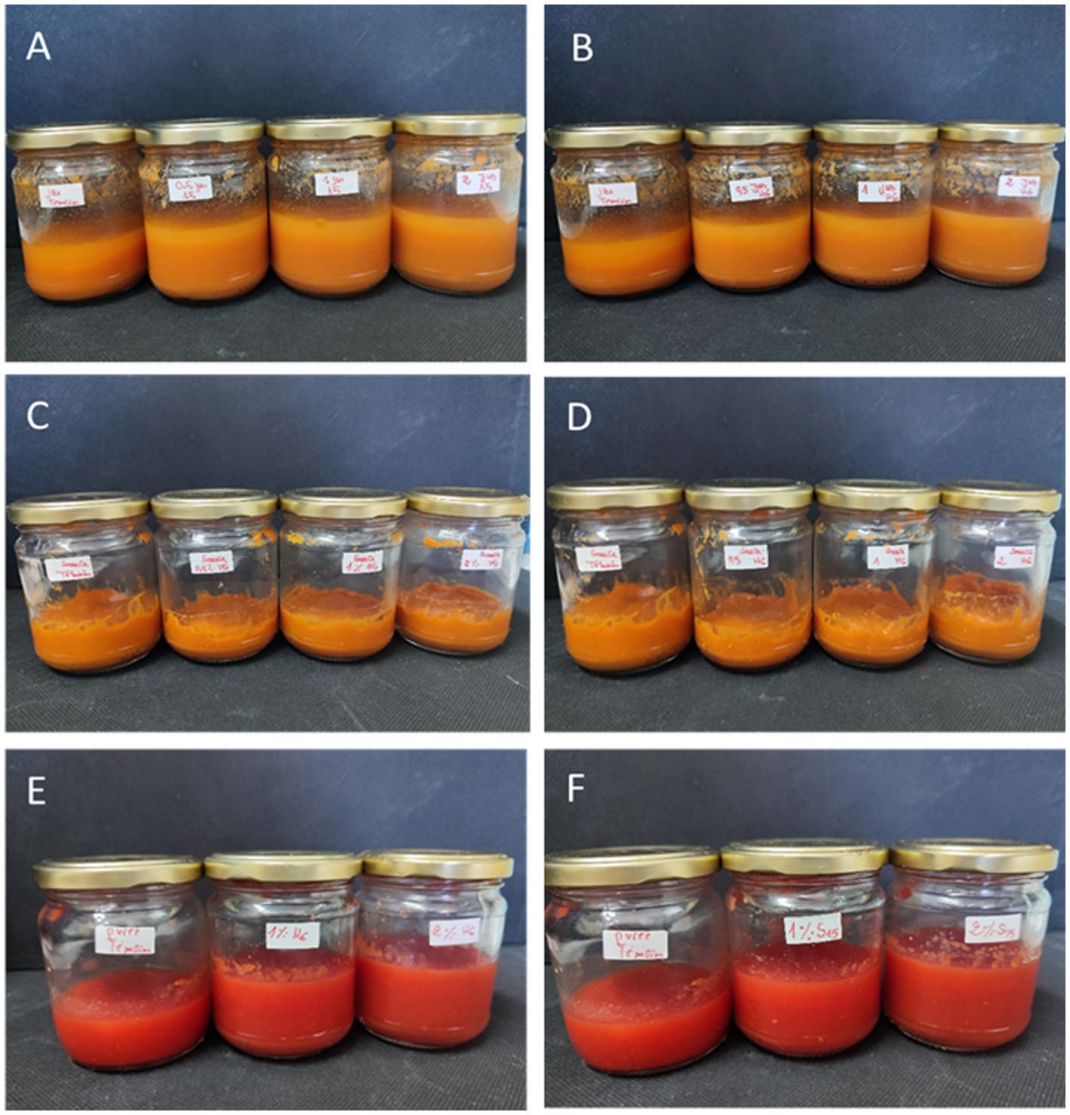
Optimization of lipopeptide concentrations for juice (**A**, H6) and (**B**, S15), sauce (**C**, H6) and (**D**, S15), and tomato puree (**E**, H6) and (**F**, S15) matrices.

**Figure 2:**
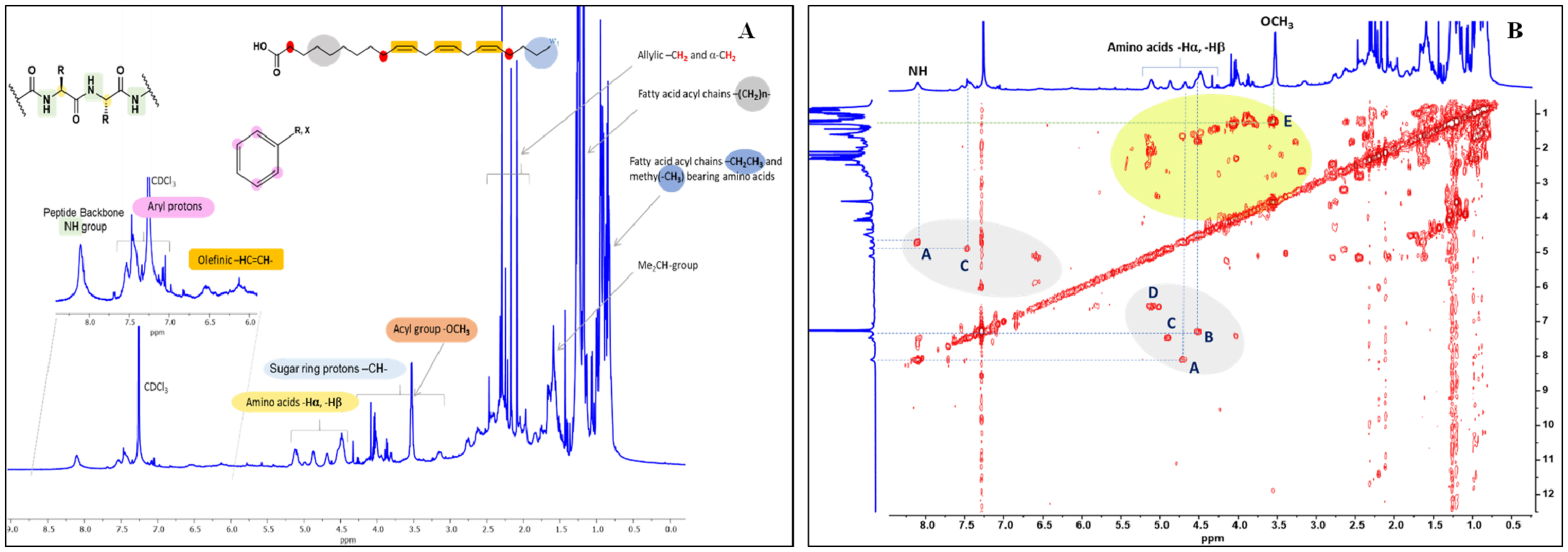
^1^H-^1^D NMR spectrum of a complex biosurfactant-lipopeptide dissolved in CDCl_3_ **(A)**. The analysis of the COSY H-H sequence in CDCl_3_ of the biosurfactant H6 in CDCl_3_, highlighting various proton correlations **(B)**.

Two-dimensional Correlation Spectroscopy (COSY) enhances structural analysis by revealing proton–proton couplings that confirm molecular connectivity (Figure 2). Specifically, correlations between amide NH protons and the α/β protons of amino acids verify the integrity of the peptide backbone. Similarly, interactions between acyl group protons and terminal methylene (-CH₂-) protons confirm the covalent attachment of fatty acid chains to the peptide moiety (31). These detailed confirmations are essential for distinguishing lipopeptides from other biosurfactants and for understanding their conformational behavior across various environments.

The amphiphilic structure elucidated by NMR is closely linked to the functional performance of these bioemulsifiers in complex food systems. Their hydrophilic peptide segments and hydrophobic lipid chains facilitate versatile interactions with food components such as proteins, polysaccharides, and pigments. These interactions contribute to improved texture, enhanced stability, and greater visual appeal (32). For example, in tomato-based products, lipopeptides help stabilize dispersed phases, reduce phase separation, and enhance the sensory quality of the product, which plays a vital role in consumer preference.

In addition to structural insights, NMR provides valuable information on dynamic molecular behaviors, helping to elucidate how factors like concentration, pH, and environmental conditions influence bioemulsifier performance (33). This level of understanding supports the rational design of lipopeptides with optimized emulsifying properties, contributing to the development of natural, sustainable, and effective emulsifiers for the food industry.

Detailed NMR analysis offers essential structural insights that connect molecular architecture to functional performance, guiding the strategic development of lipopeptide bioemulsifiers for food and biotechnological applications.

### 3.2 Effect of lipopeptides on the tomato juice microstructure

**Figure 3:**
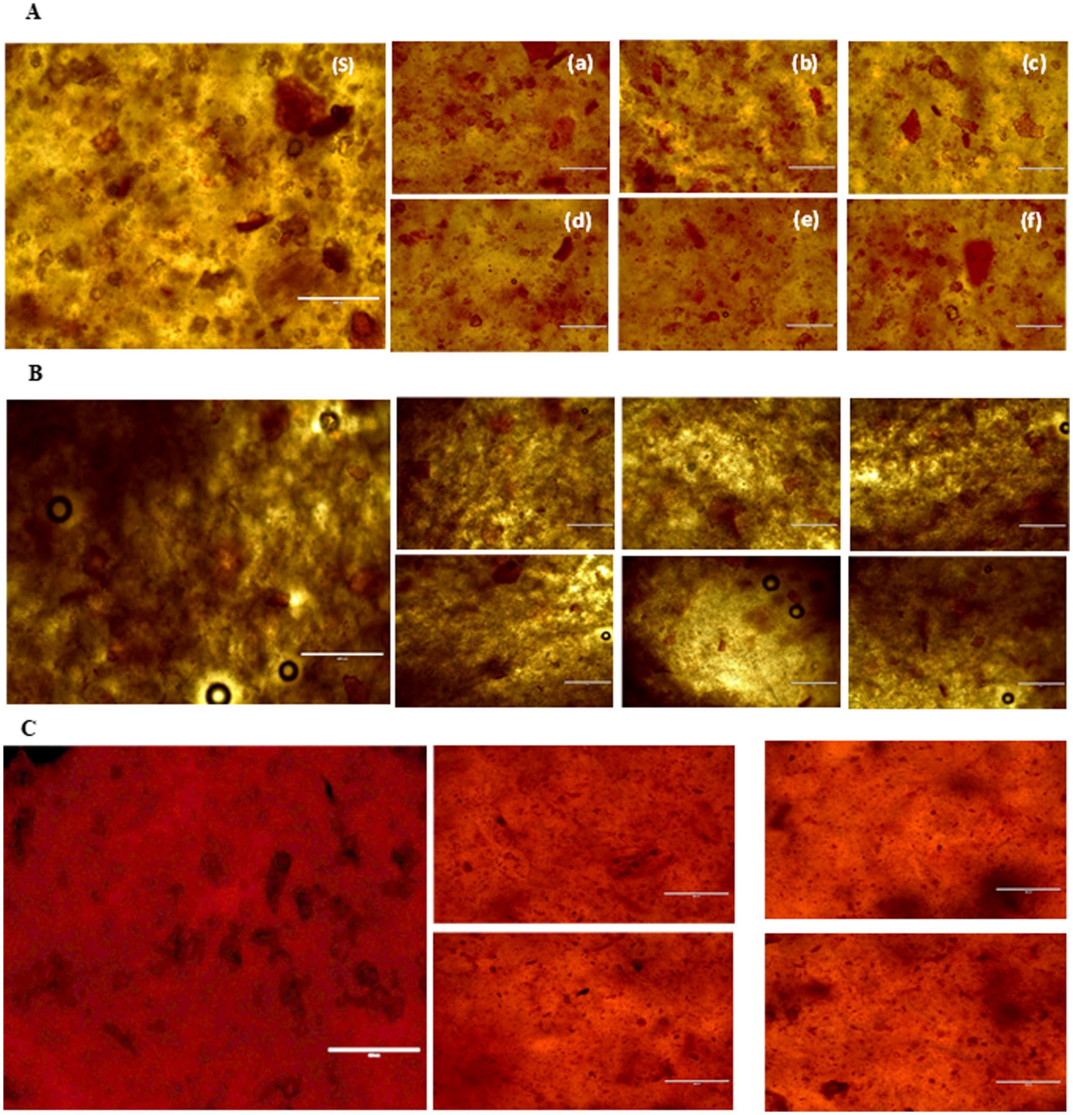
Effect of lipopeptides on the tomato juice microstructure **(A)**, the tomato sauce microstructure **(B)**, and the tomato puree microstructure **(C)**: (s) Standard without lipoppeptides; (a) – 0.5 g/L; (b) H6-1 g/L; (c) H6-2 g/L; (d) S15-0.5 g/L; (e) S15-0.51 g/L; (f) S15-2 g/L; optical microscopy. The scale bar shows 400 μm.

The impact of lipopeptide incorporation on the microstructure of tomato products (Figure 1) was assessed using optical microscopy (Figure 3). Microscopic analyses clearly demonstrate that lipopeptides significantly influence the spatial organization of tomato juice components (Figure 3A) by fostering a more interconnected network between polysaccharides and proteins. Similar effects have been reported in other food matrices, where biosurfactants act as structuring agents to improve network formation and homogeneity (34, 35). The observed densification and uniformity at higher lipopeptide concentrations (1 g/L and 2 g/L) (Figures 3B,C) align with prior findings showing that lipopeptides can enhance microstructural integrity by promoting interactions between biopolymers (36).

Microscopy images (Figure 3) corroborate these observations by revealing increased surface roughness and compactness within the tomato matrix. Comparable studies have shown that biosurfactants such as surfactin and fengycin produce a more cohesive microstructure in food emulsions and semi-solid systems, contributing to improved texture and stability (37). The role of lipopeptides as natural structuring agents is thus supported, confirming their potential to replace synthetic stabilizers in tomato-based products.

Moreover, the reduction of phase separation and enhanced dispersion of suspended particles are consistent with the amphiphilic nature of lipopeptides, which enable them to adsorb at interfaces and reduce interfacial tension (17). This steric and electrostatic stabilization has been well-documented for biosurfactants in emulsions and suspensions, resulting in improved colloidal stability and homogeneity (38).

Differences in the microstructural impact of distinct lipopeptide treatments (e.g., H6 vs. S15) warrant further investigation. Variations in molecular structure, such as fatty acid chain length or amino acid composition, can influence their interaction with pectins, proteins, and polysaccharides, thereby modulating their stabilizing efficiency and rheological behavior (39). The denser and more interconnected networks observed at higher lipopeptide concentrations, particularly for H6-treated tomato sauce (Figure 3B), suggest an enhancement of viscosity and possible gel-like behavior, consistent with biosurfactants’ known ability to alter the viscoelastic properties of food matrices (40).

These microstructural changes likely contribute to the improved texture and stability reported in other biosurfactant-enriched food products. Nonetheless, comprehensive rheological and sensory evaluations are essential to correlate these structural modifications with consumer perception and functional attributes, as recommended in recent reviews on biosurfactant applications in food systems (41).

### 3.3 Rheology analysis

The viscoelastic properties of the three types of tomato products were examined in the linear viscoelastic region by oscillatory sweep measurements of storage modulus (G′), loss modulus (G′′), and complex viscosity (η*) as a function of frequency (f). G′ reflects the elastic (solid-like) behavior of the sample, while G′′ indicates its viscous (liquid-like) behavior. Consequently, a sample with a high G′ demonstrates greater resistance to deformation, whereas a sample with a high G′′ tends to flow more readily (42).

**Figure 4:**
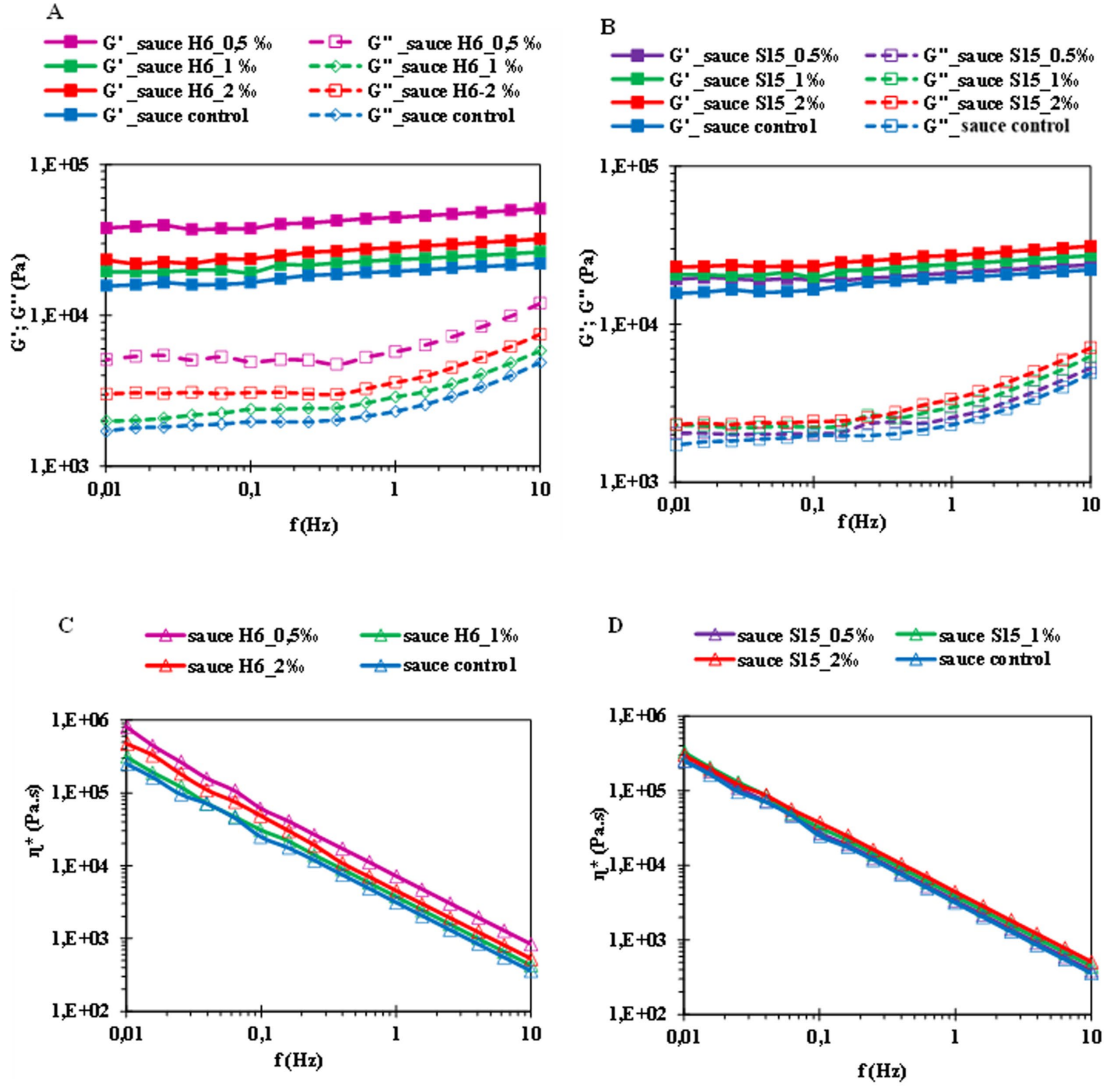
**(A,B)** G′ and G′′ moduli and **(C,D)** η* as a function of oscillation frequency for sauce prepared with and without H6 and S15, respectively.

The evolution of G′, G′′, and η* as a function of frequency for sauces prepared with and without H6 and S15 is shown in Figure 4. All sauces exhibited G′ values higher than G′′, confirming gel-like behavior (Figures 4A,B), consistent with previous reports on tomato-based products where gelation arises from pectin and other polysaccharide networks (43, 44). Higher G′ values suggest the development of a more rigid microstructure formed by interacting droplets, which have also been observed in emulsified food matrices stabilized by surfactants or biopolymers (45). This gel-like property was less pronounced for the sauce stabilized with S15, while it was most notable in the sauce stabilized with 0.5% H6. Interestingly, a moderate increase in G′ was observed with increasing S15 concentration, aligning with literature showing that biosurfactants can enhance droplet interactions and network formation (46). However, for H6 content above 0.5%, a decrease in G′ was noted, likely due to network disruption or over-saturation effects, as reported by other studies on biosurfactant concentrations affecting emulsion stability (47).

The gel-like behavior of the sauce results from various colloidal interactions, including electrostatic, steric, and van der Waals forces between droplets, which modulate the droplet interface and enable stable network formation (47). The complex viscosity of all sauces decreased with increasing frequency (Figures 4C,D), characteristic of shear-thinning fluids typical of tomato sauces and similar food emulsions (48). The sauce with 0.5% H6 showed a shift to higher viscosity values, consistent with enhanced droplet network formation at this concentration (46), whereas higher H6 concentrations reduced viscosity, possibly due to network weakening as also seen in other emulsified systems with excess surfactant (45). Conversely, S15 did not significantly affect the viscosity, suggesting different modes of interaction or molecular architecture between the two biosurfactants.

**Figure 5:**
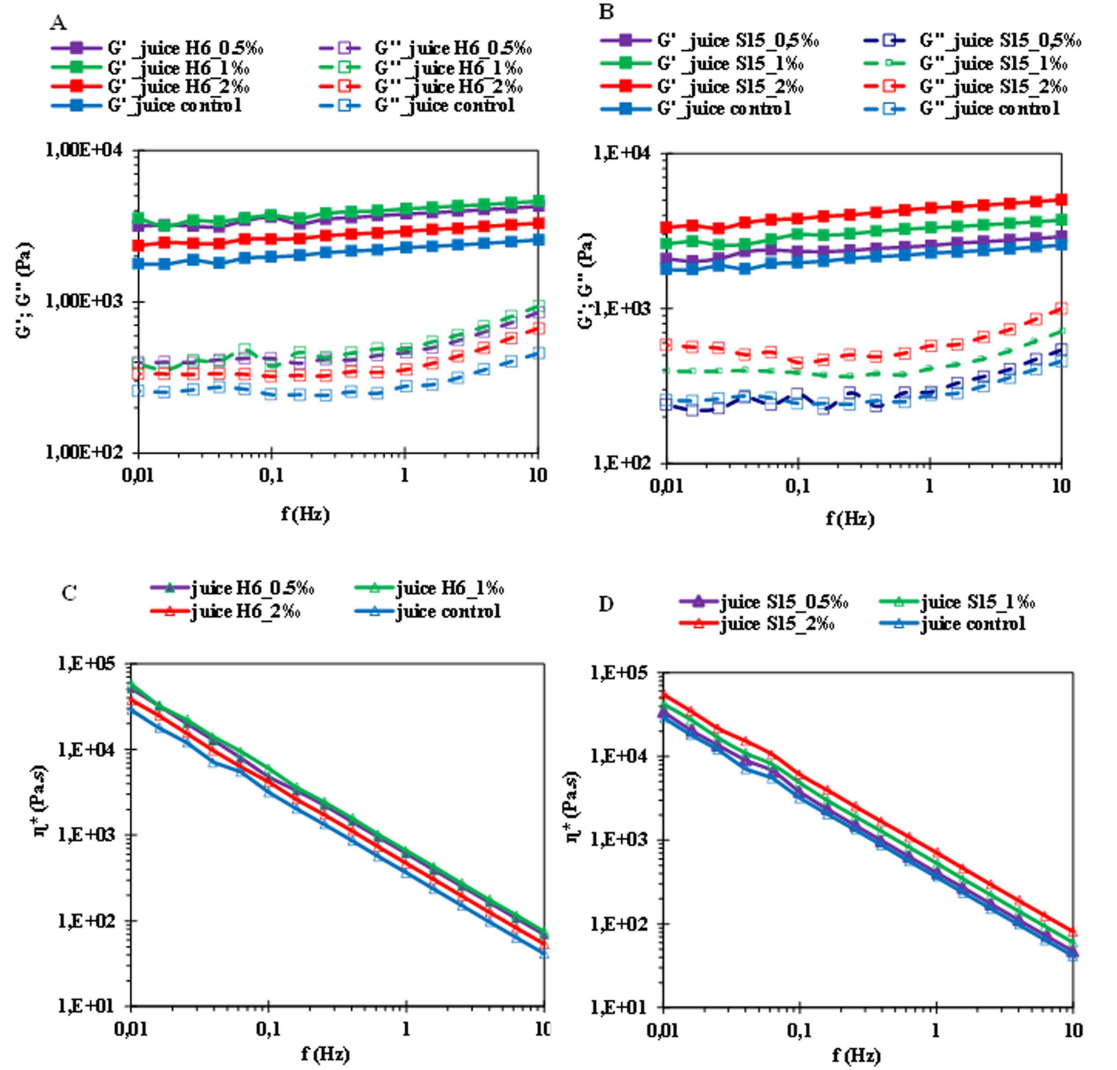
**(A,B)** G′ and G′′ moduli and **(C,D)** η* as a function of oscillation frequency for juice prepared with and without H6 and S15, respectively.

Figure 5 presents the dependence of G′, G′′, and η* versus frequency for juices prepared with varying loadings of H6 and S15. All juices exhibited shear-thinning and gel-like behavior, as commonly observed in fruit juices due to polysaccharides and suspended solids (44). The addition of S15 increased G′ and G′′ values compared to neat juice, corroborating findings by Banat et al. (2014) (36) that biosurfactants can strengthen viscoelastic properties in fluid food matrices. The presence of H6 induced a more pronounced increase at lower concentrations (0.5 and 1%), which aligns with prior observations that low doses of biosurfactants effectively enhance network formation without causing destabilization (46). The complex viscosity was also higher for juices with 2% S15 and lower concentrations of H6, indicating a more rigid microstructure formed by interacting droplets, in line with rheological studies on emulsified beverages (47).

**Figure 6:**
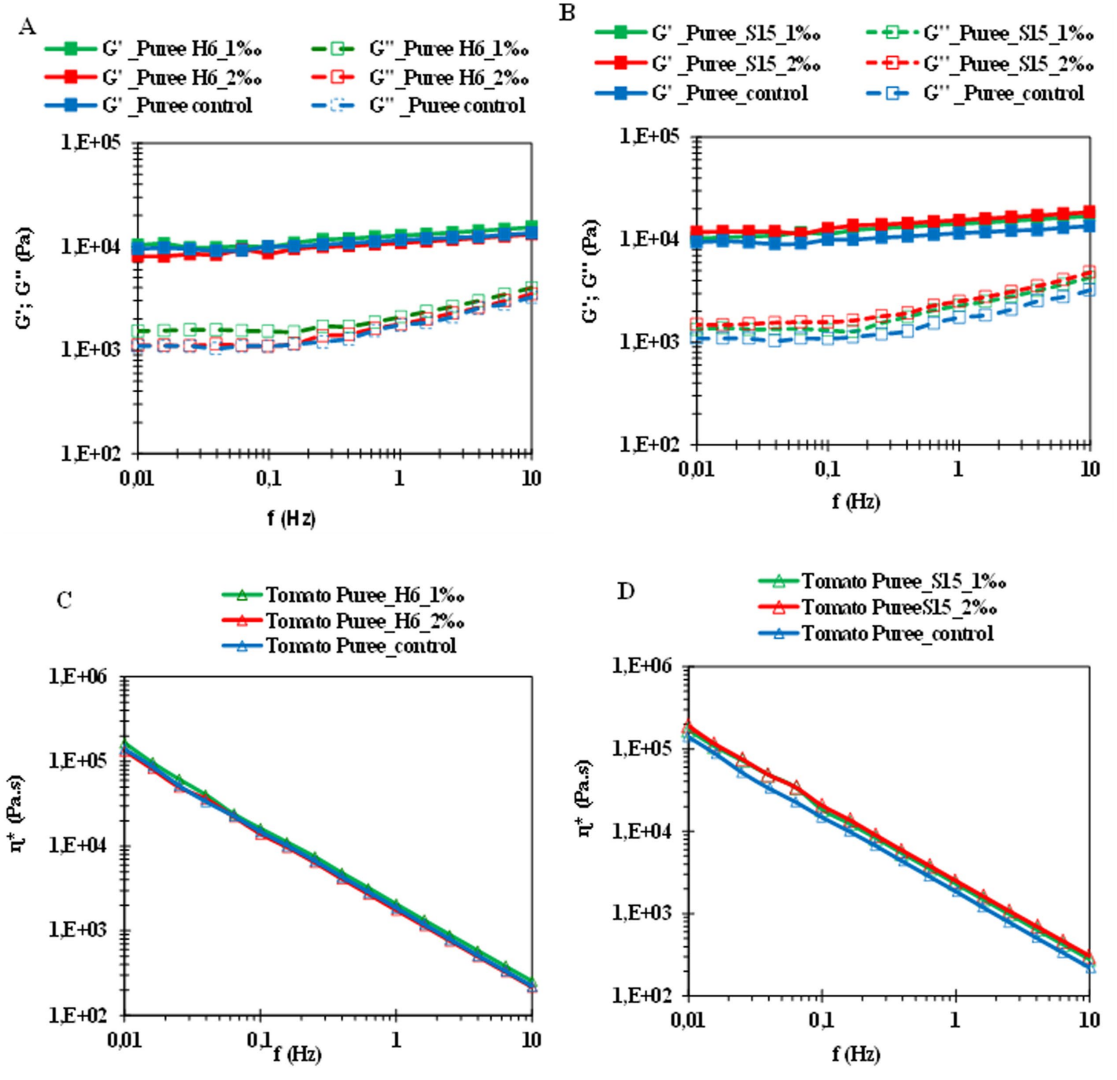
**(A,B)** G′ and G′′ moduli and **(C,D)** η* as a function of oscillation frequency for puree prepared with and without H6 and S15, respectively.

Likewise, all purées exhibited shear-thinning behavior with elastic dominance (Figure 6), consistent with the texture and composition of concentrated vegetable purées reported in literature (43). However, the presence of H6 and S15 at 1 and 2%, respectively, did not significantly alter the rheological properties of the neat sample, which suggests that the dense, complex matrix of the purée buffers the impact of added biosurfactants, as noted in previous studies where matrix composition modulates rheological response to additives (44).

The viscoelastic profiles of tomato sauce, juice, and purée supplemented with lipopeptides H6 and S15 thus reveal matrix- and concentration-dependent rheological responses. In tomato sauce and juice, H6 enhanced G′ and G′′ moduli compared to controls but showed optimal effects at low concentrations, supporting the hypothesis that low doses strengthen structural networks, while excess disrupts them (36, 46). In contrast, S15 demonstrated a more predictable, concentration-dependent increase in moduli, especially in juice, suggesting a gradual reinforcement of the viscoelastic matrix. In purée, the limited effect of both biosurfactants highlights the buffering effect of the complex matrix on texture modification (43).

Overall, these findings align with existing literature on biosurfactant applications in food matrices, where lipopeptides can act as effective natural emulsifiers and texturizers, but their impact is strongly influenced by matrix composition and concentration (45–48).

### 3.4 Viscosity measurements

The variation of the shear stress (SS) as a function of the shear rate (SR) at different temperatures (25 to 50 °C) for all the three products studied, pulp, sauce and juice, without any addition of lipopeptides and with the addition of lipopeptide H6 and S15 clearly shows a non-Newtonian behavior after a yield stress. Therefore, two models were tried to fit the experimental data the Bingham model and the classical model of Herschel–Bulkley. The parameters obtained for both models as a function of temperature are presented in Tables 1–3.

**Table 1:** Rheological parameters estimated for the pulps in study.

Product	Model	Consistency index, *K* (Pa·s^n^)	Activation energy, *Ea* kJ·mol^−1^	Yield stress, *To* (Pa)	Flow behavior index, *n*	*R^2^*	Adjusted *R^2^*
Pulp control	Bingham	0.50 ± 0.01	14.92 ± 1.25	0.43 ± 0.02	1	0.95	0.95
	Herschel-Bulkley	0.18 ± 0.02	20.12 ± 1.85	0.26 ± 0.02	0.64 ± 0.03	0.98	0.98
Pulp H6	Bingham	0.11 ± 0.01	−6.69 ± 3.61	1.94 ± 0.09	1	0.84	0.84
	Herschel-Bulkley	1.20 ± 0.27	−9.57 ± 4.15	0.87 ± 0.25	0.35 ± 0.05	0.84	0.84
Pulp S15	Bingham	0.098 ± 0.01	37.15 ± 5.71	1.09 ± 0.10	1	0.86	0.86
	Herschel-Bulkley	0.35 ± 0.12	54.74 ± 9.54	0.82 ± 0.16	0.63 ± 0.10	0.88	0.87

**Table 2:** Rheological parameters for the sauces in study.

		Parameters					
Product	Model	Consistency index, *K* (Pa·s^n^)	Activation energy, *Ea* kJ·mol^−1^	Yield stress, *To* (Pa)	Flow behavior index, *n*	*R^2^*	Adjusted *R^2^*
Sauce control	Bingham	2.82 ± 0.10	22.87 ± 1.95	7.20 ± 0.13	1	0.94	0.94
	Herschel-Bulkley	6.03 ± 0.45	16.93 ± 1.44	4.62 ± 0.40	0.48 ± 0.04	0.99	0.98
Sauce H6	Bingham	3.44 ± 0.23	18.62 ± 3.01	4.98 ± 0.22	1	0.78	0.78
	Herschel-Bulkley	6.16 ± 1.49	26.93 ± 4.41	2.57 ± 1.40	0.47 ± 0.14	0.80	0.79
Sauce S15	Bingham	3.68 ± 0.17	13.14 ± 2.05	4.52 ± 0.17	1	0.87	0.86
	Herschel-Bulkley	4.68 ± 0.65	15.22 ± 2.56	3.69 ± 0.58	0.73 ± 0.13	0.87	0.87

**Table 3:** Rheological parameters for the juices in the study.

Product	Model	Consistency index, *K* (Pa·s^n^)	Activation energy, *Ea* kJ·mol^−1^	Yield stress, *To* (Pa)	*R^2^*	Adjusted *R^2^*
Juice control	Power law	1.36 ± 0.08	−11.47 ± 7.17	0.24 ± 0.02	0.98	0.98
Juice H6	Power law	1.35 ± 0.08	12.78 ± 6.63	0.23 ± 0.01	0.99	0.98
Juice S15	Power law	1.43 ± 0.11	54.50 ± 9.30	0.22 ± 0.02	0.97	0.97

The friction and other interactions between the insoluble particles dispersed by the pulp are related to the consistency index value (*K*), and the behavior index (*n*) gives information on the difficulty for the particle to align with the flowing direction: the closer to 1.0 the less expressive this “alignment effect” is. The presence of yield stress is a typical behavior of multiphase materials such as pulp juice and sauce prepared from vegetables and fruits composed of a dispersion of insoluble components such as fragments of cellular walls and even small pieces of peels in a water solution (usually a serum containing sugars, minerals, proteins, and soluble polysaccharides) (49). The same behavior was observed for mango acerola juice (50), siriguela pulp (51), açai pulp (52, 53), Indian coffee plum (*Flacourtia jangomas*) pulp (54) and jaboticaba pulp (55). Ahmed et al. (56) reported that emergent preservation technologies such as high-pressure, high-pressure homogenization, and thermosonication change the rheological properties of, among other products, tomato juice or tomato paste. Augusto et al. (57) reported that tomato juice shows various behaviors, Newtonian, shear-thinning, with yield stress or thixotropic behavior depending on the processing procedure such as homogenization and Ribeiro et al. (58) observed a similar trend in mango. For all the tomato products that were studied, it could be seen that the Bingham model never fitted the experimental data better than the Herschel-Buckley (Supplementary data 1–3). This proves that n was always <1 and therefore that the alignment effect was expressive for all products, with special emphasis for the ones where the lipopeptides H6 were added, since “n” was lower.

The yield stress (SS_0_) in the studied products is mainly caused by a higher inter-particle attraction that forms a network. It is defined as the minimum shear stress required to initiate the flow of a product at the moment when this network is broken (59). Processing such products implies a resistance to flowing to a certain level of shear stress, affecting the design of the process, the equipment, or even the product. The higher the activation energy, the higher the sensitivity of apparent viscosity to temperature variation (59).

It appears that yield stress increases while the consistency index decreases with increasing temperature. Concerning the flow index, this is nearly constant except for the highest temperature. As we shall see later, this behavior could be explained by enhanced interactions between granules of the sludge suspension.

### 3.5 Color variation of tomato product formulations

Color is a key quality attribute influencing consumer perception and acceptability of tomato-based products (60). In this section, we examine how the incorporation of lipopeptides from *Bacillus* strains H6 and S15 affects the colorimetric parameters in tomato juice, sauce, and pulp formulations (Table 4).

**Table 4:** Color parameters (L*, a*, b*, c*, h*, ΔE, a*/b*) of tomato products with different lipoppeptides concentrations.

Sample	L*	a*	b*	c*	h*	ΔE	a*/b*
Tomato juice CS	44.68 ± 0.02	18.09 ± 0.12	21.10 ± 0.03	27.79 ± 0.09	49.40 ± 0.15	0.00	0.86
0.5 g/kg H6	46.67 ± 0.00	19.20 ± 0.04	23.32 ± 0.01	30.20 ± 0.03	50.52 ± 0.05	3.18 ± 0.06	0.81
1 g/kg H6	46.84 ± 0.01	18.91 ± 0.06	23.39 ± 0.00	30.08 ± 0.04	51.05 ± 0.09	3.25 ± 0.07	0.81
2 g/kg H6	47.78 ± 0.04	19.08 ± 0.01	24.30 ± 0.02	30.89 ± 0.02	51.86 ± 0.03	4.56 ± 0.05	0.79
0.5 g/kg S15	46.67 ± 0.04	19.48 ± 0.18	23.40 ± 0.06	30.45 ± 0.15	50.22 ± 0.22	3.34 ± 0.20	0.83
1 g/kg S15	46.77 ± 0.03	19.29 ± 0.09	23.25 ± 0.04	30.21 ± 0.08	50.31 ± 0.08	3.23 ± 0.10	0.83
2 g/kg S15	46.83 ± 0.01	17.87 ± 0.20	23.49 ± 0.05	29.52 ± 0.15	52.73 ± 0.28	3.22 ± 0.22	0.76
Tomato sauce CS	47.34 ± 0.02	24.44 ± 0.02	26.40 ± 0.04	35.96 ± 0.03	47.22 ± 0.04	8.69 ± 0.10	0.93
0.5 g/kg H6	47.61 ± 0.01	24.33 ± 0.02	26.98 ± 0.09	36.33 ± 0.07	47.96 ± 0.09	9.06 ± 0.11	0.90
1 g/kg H6	48.22 ± 0.01	24.50 ± 0.02	27.54 ± 0.04	36.86 ± 0.03	48.34 ± 0.05	9.75 ± 0.07	0.89
2 g/kg H6	48.11 ± 0.04	24.21 ± 0.03	27.55 ± 0.04	36.67 ± 0.05	48.68 ± 0.02	9.53 ± 0.05	0.88
0.5 g/kg S15	47.77 ± 0.02	24.32 ± 0.01	26.92 ± 0.05	36.29 ± 0.05	47.9 ± 0.04	9.07 ± 0.06	0.90
1 g/kg S15	47.83 ± 0.03	24.36 ± 0.02	27.31 ± 0.03	36.6 ± 0.04	48.26 ± 0.02	9.37 ± 0.05	0.89
2 g/kg S15	48.00 ± 0.02	24.13 ± 0.02	27.26 ± 0.01	36.41 ± 0.00	48.48 ± 0.05	9.24 ± 0.06	0.89
Tomato Pulpe CS	36.54 ± 0.00	14.80 ± 0.03	09.33 ± 0.04	17.50 ± 0.03	32.24 ± 0.10	14.68 ± 0.12	1.59
1 g/kg H6	37.54 ± 0.01	17.93 ± 0.05	10.47 ± 0.04	20.77 ± 0.07	30.28 ± 0.03	12.81 ± 0.09	1.71
2 g/kg H6	38.25 ± 0.02	18.30 ± 0.02	11.12 ± 0.01	21.41 ± 0.02	31.28 ± 0.02	11.87 ± 0.05	1.65
1 g/kg S15	37.26 ± 0.01	16.83 ± 0.06	10.29 ± 0.05	19.72 ± 0.06	31.44 ± 0.16	13.17 ± 0.11	1.64
2 g/kg S15	37.63 ± 0.02	17.94 ± 0.01	10.84 ± 0.05	20.97 ± 0.03	31.14 ± 0.12	12.45 ± 0.09	1.65

The control sample (CS) had an L* value of 44.68, while samples treated with H6 showed increased lightness, especially at 2 g/kg (L* = 47.80). In contrast, samples with S15 had slightly lower L* values (e.g., 46.83 for 2 g/kg S15), indicating a darker appearance. The a* values, representing redness, were highest in the control and generally decreased with treatment, particularly with S15. Similarly, the b* values and chroma (C*) were also higher in H6-treated samples compared to those treated with S15, reflecting stronger yellow tones. The hue angle (h°) increased slightly with treatment, indicating a shift toward yellowish-red. The color difference (ΔE) values were higher in 2 g/kg H6 (4.56) and S15 (3.22) samples, suggesting noticeable visual differences from the control. The a*/b* ratio, reflecting red to yellow dominance, decreased slightly in all treatments, particularly with higher S15 concentrations.

Tomato sauce samples showed higher lightness compared to juice, with L* values reaching 48.22 in the 1 g/kg H6 group. The redness (a*) and yellowness (b*) parameters increased in all treated samples compared to the control. Furthermore, 0.5 g/kg H6 and S15 treatments caused the highest a* values (24.33 and 24.36, respectively), suggesting an enhancement in red coloration. The ΔE values for all treated samples were above 8, which indicates significant perceptual color differences from the control. Overall, the a*/b* ratios in sauces remained relatively high (0.89–0.93), demonstrating strong red pigmentation stability.

The pulp samples had lower a* and b* values compared to juice and sauce, likely due to matrix differences. The L* values increased with treatment, with the highest lightness in the 1 g/kg H6 sample (37.54). The ΔE values in pulp were markedly higher than in juice and sauce, exceeding 12 in all treated samples. This suggests that pulp was the most visually impacted product in terms of color. Among all treatments, the highest ΔE was observed in the 1 g/kg H6 sample (ΔE = 13.17), indicating the most visible color change from the control.

Among all product types, tomato sauce exhibited the most stable color properties with respect to a*/b* ratio and chroma. H6-treated samples generally resulted in higher chroma and lower hue angles, indicating more saturated and redder colors than S15. On the other hand, S15 treatments showed to decrease redness (a*) and increase hue angle, especially at higher concentrations, leading to a shift toward more yellowish tones. Across all matrices, the 1 g/kg H6 concentration was the most effective in enhancing brightness and chroma without significantly compromising redness.

## 4 Conclusion

Lipopeptides from *Bacillus* strains H6 and S15 show strong potential as natural emulsifiers and texturizers for tomato-based products. Their amphiphilic structure enables improved microstructure, enhanced viscoelasticity, and better color characteristics, particularly with H6 at 0.5–1 g/kg. Rheological and color analyses confirmed their ability to stabilize food matrices and enhance visual appeal. However, excessive concentrations may reduce stability, highlighting the need for dose optimization. These findings support their application as sustainable alternatives to synthetic additives in clean-label food formulations.

## Data Availability

The datasets presented in this study can be found in online repositories. The names of the repository/repositories and accession number(s) can be found in the article/Supplementary material.
