# Supplementary material for: Bacterial lipopeptides from Bacillus: natural biostructuring agents for improving texture and stability in tomato-based functional foods
Source: Front Nutr. 2026 Jun 8;13:1830310. doi: 10.3389/fnut.2026.1830310 (PMC13283810; doi:10.3389/fnut.2026.1830310)
Supplement: Supplementary file 1 [file Table_1.docx]

| 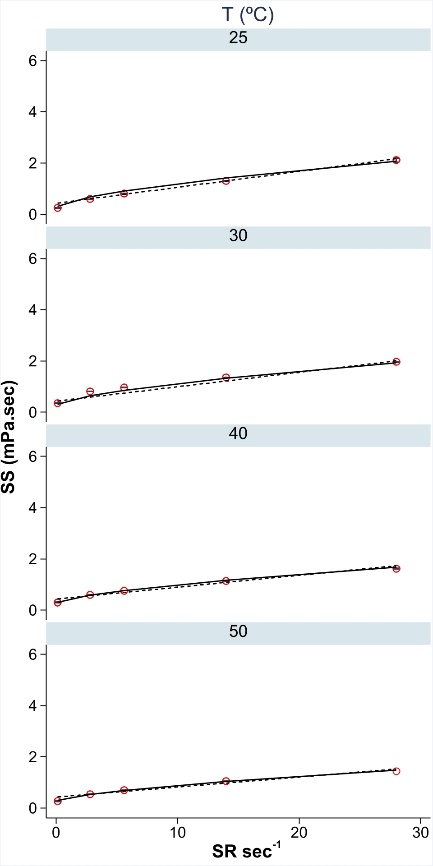 | 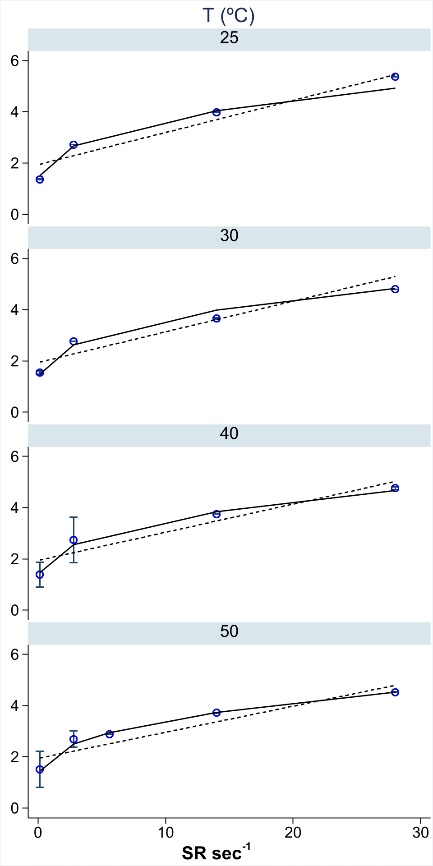 | 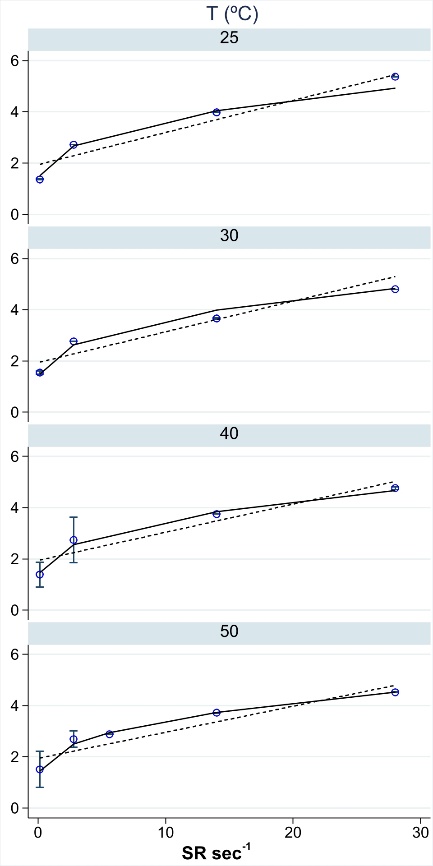 |
| --- | --- | --- |

**Supplementary data 1** - Pulps’ behavior with temperature effect. **ο** – sauce (control), **ο**- sauce with H6 lipopeptides and **ο** – sauce with S15 lipopeptides

| 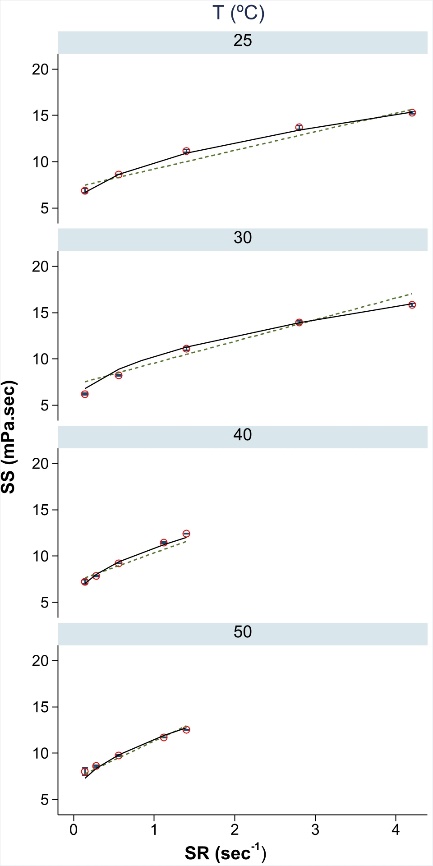 | 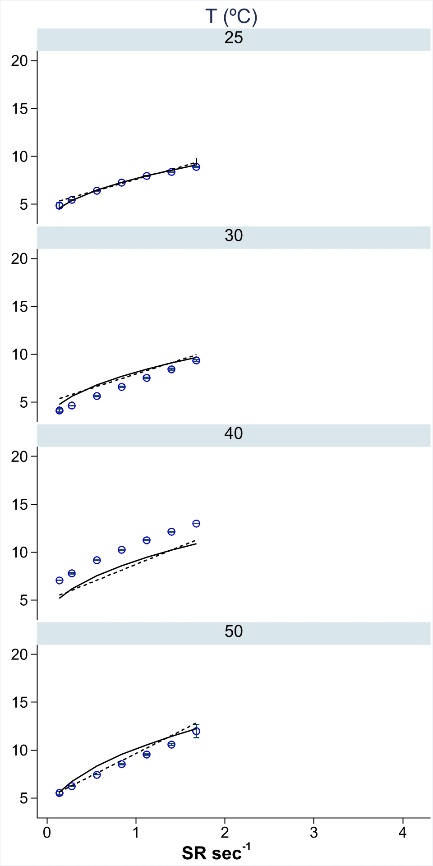 | 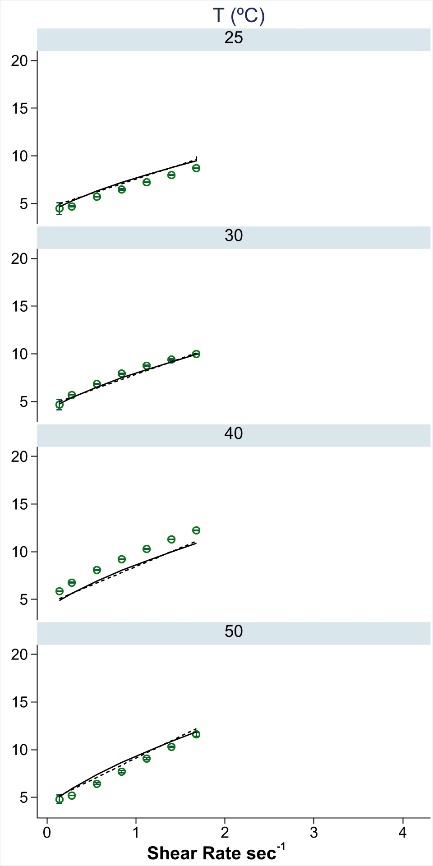 |
| --- | --- | --- |

**Supplementary data 2** - Sauces’ behavior with temperature effect. **ο** – sauce (control), **ο**- sauce with h6 lipopeptides and **ο** – sauce with s15 lipopeptides. Full line – Herchel Prediction by Buckley model, Dashed line prediction by Bingham model

| 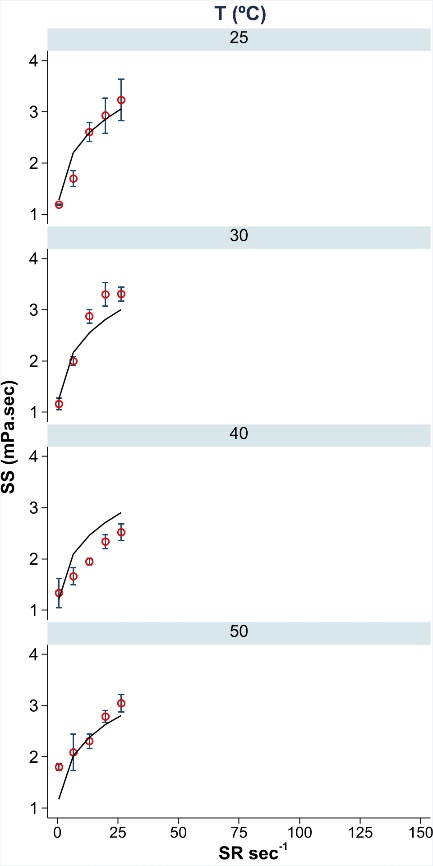 | 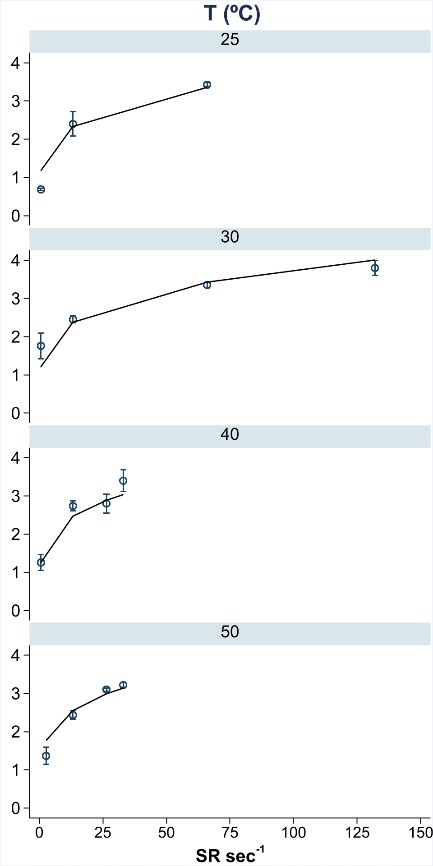 | 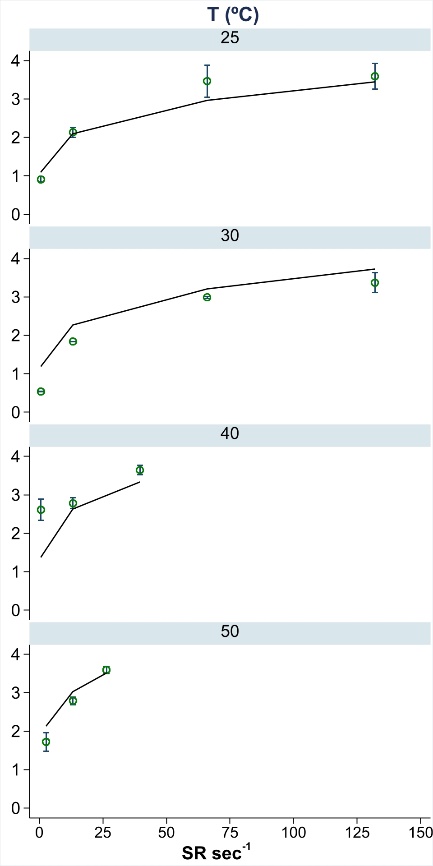 |
| --- | --- | --- |

| \|  \|  \|  \| \| --- \| --- \| --- \| |  |  |
| --- | --- | --- | --- | --- | --- |

**Supplementary data 3**- Juice’s behavior with temperature effect. **ο** – sauce (control), **ο**- sauce with H6 lipopeptides, and **ο** – sauce with S15 lipopeptides

| Pulp | Sauce | Juice |
| --- | --- | --- |
| 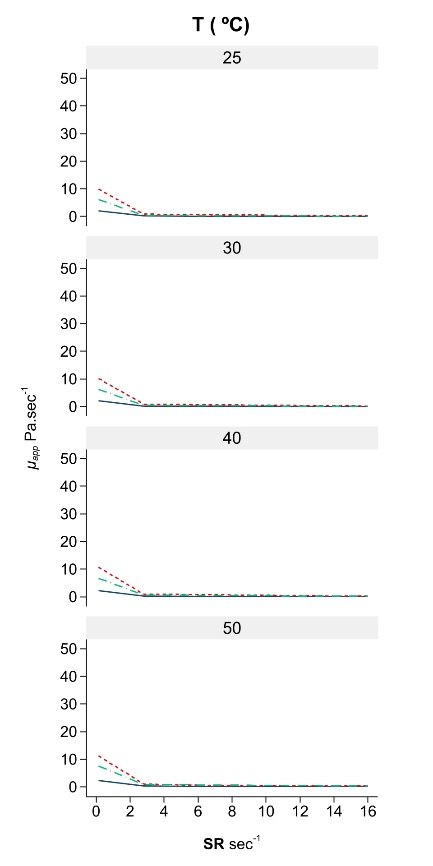 | 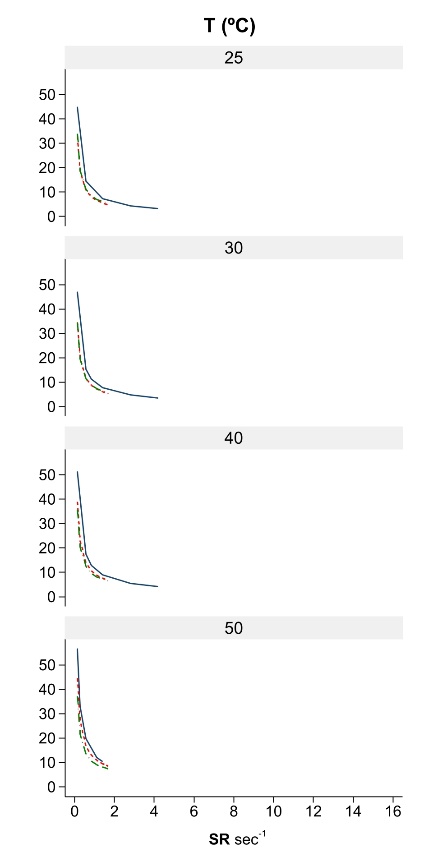 | 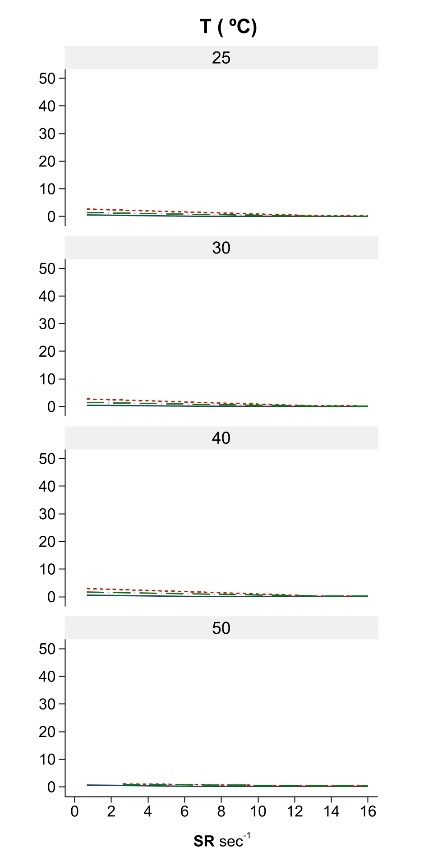 |

**Supplementary data 4**- Apparent viscosity of pulp, sauce, and juice predicted by the Herschel-Buckley model for pulp and sauce and by the power-law model for the juice. Control product ‒; product +lipopeptides h6 ⋅⋅⋅; product with lipopeptides s15 ⋅‒⋅;
